# The potential of hybrid breeding to enhance leaf rust and stripe rust resistance in wheat

**DOI:** 10.1007/s00122-020-03588-y

**Published:** 2020-04-12

**Authors:** Ulrike Beukert, Guozheng Liu, Patrick Thorwarth, Philipp H. G. Boeven, C. Friedrich H. Longin, Yusheng Zhao, Martin Ganal, Albrecht Serfling, Frank Ordon, Jochen C. Reif

**Affiliations:** 1 Institute for Resistance Research and Stress Tolerance, Julius Kuehn-Institute (JKI), Erwin-Baur-Straße 27, 06484 Quedlinburg, Germany; 2 BASF Agricultural Solutions Belgium NV, Technologiepark 101, 9052 Zwijnaarde, Ghent, Belgium; 3 State Plant Breeding Institute, University of Hohenheim, Fruwirthstraße 21, 70593 Stuttgart, Germany; 4 Leibniz Institute of Plant Genetics and Crop Plant Research (IPK), Corrensstraße 3, 06466 Stadt Seeland OT Gatersleben, Germany; 5 TraitGenetics GmbH, Am Schwabeplan 1b, 06466 Stadt Seeland, OT Gatersleben, Germany

## Abstract

**Key message:**

Hybrid wheat breeding is a promising strategy to improve the level of leaf rust and stripe rust resistance in wheat.

**Abstract:**

Leaf rust and stripe rust belong to the most important fungal diseases in wheat production. Due to a dynamic development of new virulent races, epidemics appear in high frequency and causes significant losses in grain yield and quality. Therefore, research is needed to develop strategies to breed wheat varieties carrying highly efficient resistances. Stacking of dominant resistance genes through hybrid breeding is such an approach. Within this study, we investigated the genetic architecture of leaf rust and stripe rust resistance of 1750 wheat hybrids and their 230 parental lines using a genome-wide association study. We observed on average a lower rust susceptibility for hybrids in comparison to their parental inbred lines and some hybrids outperformed their better parent with up to 56%. Marker-trait associations were identified on chromosome 3D and 4A for leaf rust and on chromosome 2A, 2B, and 6A for stripe rust resistance by using a genome-wide association study with a Bonferroni-corrected threshold of *P* < 0.10. Detected loci on chromosomes 4A and 2A were located within previously reported genomic regions affecting leaf rust and stripe rust resistance, respectively. The degree of dominance was for most associations favorable in the direction of improved resistance. Thus, resistance can be increased in hybrid wheat breeding by fixing complementary leaf rust and stripe rust resistance genes with desired dominance effects in opposite parental pools.

**Electronic supplementary material:**

The online version of this article (10.1007/s00122-020-03588-y) contains supplementary material, which is available to authorized users.

## Introduction

Wheat (*Triticum aestivum* L.) is an important crop in the world with an annual production of ~ 770 million tons harvested on ~ 220 million hectares in 2017 (FAO 2019). Wheat is a central source of calories and proteins for human nutrition and plays therefore an important role to feed the earth´s growing population (Peña-Bautista et al. 2017). Leaf rust caused by *Puccinia triticina* and stripe rust caused by *Puccinia striiformis* f. sp. *tritici* belong to the most important fungal diseases of wheat (Huerta-Espino et al. 2011; Wellings 2011). Leaf rust and stripe rust show enormous genetic diversity due to phases of sexual recombination within their life cycle. The naturally occurring rust population is subject of local adaption, stepwise evolution, and a high selection pressure leading to the dynamic development of new pathotypes (Bolton et al. 2008; Schwessinger 2017). Therefore, epidemics appear in high frequency resulting in yield losses up to 70% (Chen 2005; Huerta-Espino et al. 2011) as well as a reduced grain quality (Prescott et al. 1986).

The use of effective resistance genes against rust diseases in wheat cultivars is a sustainable and environment-friendly solution to reduce yield and quality losses. Currently there are around 90 and 80 resistance genes for leaf rust (*Lr*-genes) and stripe rust (*Yr*-genes) known, respectively (McIntosh et al. 1995, 2005, 2009, 2013, 2015, 2017). The majority of those cause race-specific resistance mostly leading to hypersensitive cell death (Bolton et al. 2008; Singh et al. 2005). In contrast to this, very few genes are known, which are non-race-specific and result in a quantitative reduction in the infection level at the adult plant stage like *Lr34* (Bolton et al. 2008), *Yr18*, or *Yr29* (Chen 2013). Race-specific resistance causes complete resistance at the seedling stage and is based on a single gene, which can be easily overcome by fast developing rust populations (Bolton et al. 2008; Schwessinger 2017).

To promote the effectiveness of resistance, breeders aim to pyramidize resistance genes within the same cultivar (Singh et al. 2005). Stacking dominant resistance genes can be most efficiently implemented through hybrid breeding (Longin et al. 2012). Currently, hybrid breeding is implemented in several wheat breeding programs, because it promises to boost yield and yield stability (Jiang et al. 2017; Longin et al. 2013; Mühleisen et al. 2014). The potential of stacking resistance genes via hybrid breeding strongly depends on the frequency of genes displaying complete dominance. Nevertheless, there is a lack of background knowledge on the genetic architecture and degree of dominance of resistance against rust diseases in wheat.

Our study is based on a comprehensive hybrid wheat population including 1750 hybrids derived from crossing of 189 female and 41 male lines using an incomplete factorial mating design. The parents and hybrids were evaluated in multi-environment field trials for leaf rust and stripe rust resistance and genotyped using a 15 k SNP array. The objectives of this study were to (1) identify single nucleotide polymorphisms (SNPs) associated with adult plant resistance against leaf rust and stripe rust by performing genome-wide association mapping, (2) investigate the genetic architecture plus the degree of dominance of leaf rust and stripe rust resistance within a European hybrid wheat population, and (3) evaluate the resistance improvement based on a hybrid breeding strategy.

## Materials and methods

### Plant material

This study comprised 189 female and 41 male elite winter wheat lines and their 1750 single-cross hybrids, which were generated following an incomplete factorial mating design using chemical hybridization agents. The elite lines were provided by the following 13 wheat breeding companies: BASF Agricultural Solutions GmbH, Deutsche Saatveredelung AG, KWS LOCHOW GmbH, Limagrain GmbH, Pflanzenzucht Oberlimpurg, RAGT-Saaten GmbH, Saatzucht Bauer GmbH, Saatzucht Josef Breun GmbH & Co. KG, Saatzucht Streng-Engelen GmbH & Co. KG, Secobra Saatzucht GmbH, Strube Research GmbH & Co. KG, Syngenta Seeds GmbH, and W. von Borries-Eckendorf GmbH & Co. KG. Details on the crossing design have been described elsewhere (Boeven et al. 2020).

### Field trials and experimental design

Hybrids and their parental lines were grown in multi-location, unreplicated field trials in two years (2016, 2017) to monitor their leaf rust and stripe rust severity. Leaf rust data were collected in seven different environments while stripe rust was observed in five environments (Table 1).

**Table 1:** Characterization of environments, in which leaf rust and stripe rust severity were evaluated

Location	Year	Latitude	Longitude	Soil type	Leaf rust	Stripe rust
Asendorf	2016	52.738315	9.006696	Luvisol		Yes
Feldkirchen	2017	48.29080	11.55063	Cambisol	Yes	
Hadmersleben	2016	51.5837	11.1751	Deep loam	Yes	Yes
Hadmersleben	2017	51.5837	11.1751	Deep loam	Yes	Yes
Leopoldshöhe	2017	51.58456	8.40396	Luvisol	Yes	
Northeim	2016	51.44240	9.54367	Loess-/Loamsoil	Yes	
Rosenthal	2016	52.181889	10.105288	Chernozem	Yes	Yes
Söllingen	2017	52.097285	10.925914	Chernozem	Yes	Yes

The field trials were unreplicated and included the same 11 checks (*JB Asano*, *Julius*, *RGT Reform*, *Colonia*, *KWS Loft*, *Rumor*, *Tobak*, *Elixer*, *Hybred*, *Hystar*, *LG Alpha*) for every environment. Field trials were randomized following an α-design. Infection of genotypes with leaf rust and stripe rust occurred naturally and was scored at the date of flowering (EC stage 65) on the flag leaf. An ordinal scale from 1 to 9 on the basis of the Bundessortenamt (2000) was used in order to score infections, where one stands for minimal symptoms and nine indicates extensive disease symptoms.

### Analysis of phenotypic data

Detection of outliers and estimation of variance components was conducted implementing the following mixed linear model:

$$y_{ijkl} = \mu + e_{l} + b_{k|l} + g_{ij} + m_{i} + f_{j} + s_{ij} + \left( {me} \right)_{il} + \left( {fe} \right)_{jl} + \varepsilon_{ijkl} ,$$

where $y_{ijkl}$ is the performance of lines $(i = j)$ or hybrids $(i \ne j)$ arising from a cross between the *i*th parent with the *j*th parent in the *k*th incomplete block in the *l*th environment. $\mu$ refers to the overall population mean. $e_{l}$ is the effect of the *l*th environment, i.e., location by year combination, $b_{k|l}$ represents the block effect of the *k*th block nested within the *l*th environment.$g_{ij}$ was only modeled for the parental lines and stands for their genotypic effect. $m_{i}$ and $f_{j}$ were modeled for hybrids and are the GCA effects of the *i*th and *j*th of the male and female parent, respectively, $s_{ij}$ symbolizes the SCA effect of the cross between the *i*th and *j*th parents. $\left( {me} \right)_{il}$ as well as $\left( {fe} \right)_{jl}$ which were only modeled for hybrids, are the interaction between the GCA effect of the *i*th and *j*th parent with the *l*th environment., $\varepsilon_{ijkl }$ refers to the corresponding residuals. All effects except the intercept were modeled as random effects.

A second model was used to obtain best linear unbiased estimations (BLUEs) across environments.

$$y_{ikl} = \mu + g_{i} + e_{l} + b_{k|l} + \varepsilon_{ikl} ,$$

where $y_{ikl}$ is the phenotypic observation of the *i*th genotype in the *k*th block at the *l*th environment. $\mu$ is the intercept, $g_{i}$ symbolizes the genotypic effect of the *i*th individual and $e_{l}$ stands for the effect of the *l*th environment. $b_{k|l}$ represents the block effect of the *k*th block nested within the *l*th environment, while $\varepsilon_{ikl}$ is the residual error associated with the observation $y_{ikl}$. The genotype effect was assumed as fixed to estimate the BLUEs, while all remaining effects were treated as random. Broad-sense heritability was calculated using variance component estimates of the first model as:

$$h^{2} = \frac{{\sigma_{\text{Genotype}}^{2} }}{{\sigma_{\text{Phenotype}}^{2} }} = \frac{{\sigma_{\text{Genotype}}^{2} }}{{\sigma_{\text{Genotype}}^{2} + \frac{{\sigma_{G \times E}^{2} }}{{{\text{No}} .\;{\text{of}}\;{\text{environment}}}} + \frac{{\sigma_{\text{error}}^{2} }}{{{\text{No}} .\;{\text{of}}\;{\text{environment}}}}}}$$

Variance of genotypes was estimated as the sum of variance components of GCA and SCA effects. Variance of interaction effects of genotypes and environments were estimated as the sum of variance of GCA-by-environment interaction effects. Heritability for single locations was estimated following the concept of genomic heritability to evaluate the quality of field trials. This was done by estimating the prediction abilities applying fivefold cross-validation as outlined in detail elsewhere (Schulthess et al. 2018).

### Genotypic analysis

The extraction of DNA was conducted in compliance with known standard procedures (Stein et al. 2001). Parental lines were genotyped using a 15 k single-nucleotide-polymorphism (SNP) array containing a subset of the wheat 90 k Illumina Infinium array (Wang et al. 2014). Composition of the 15 k SNP chip and the genotyping was implemented by TraitGenetics GmbH (http://www.traitgenetics.com). Population structure of the parental pools was examined by calculating their Rogers’ distances in addition to perform a principle coordinate analysis (PCoA). Genotypic information was imputed in accordance to He et al. (2015). Quality filtering was performed whereby redundant markers, markers with missing values > 5%, heterozygosity of > 5% in inbred material, or a minor allele frequency (MAF) of < 5% were excluded. After this selection, 10,453 markers and 1768 genotypes of high quality were left and used for association mapping. The procedure for the association mapping was previously described in detail by Liu et al. (2016). We applied the following model:

$$Y = \mu + Aa + Dd + Zz + \varepsilon ,$$

where *Y* describes BLUEs across the locations, $\mu$ is the vector of intercept effects, *a* symbolizes the vector of additive effects, *d* is a vector of dominance effects, *z* is representing the vector of polygene background effects and $\varepsilon$ stands for the vector of residual effects. *A*, *D*, and *Z* were incidence matrices, which relates the BLUEs to the vectors *a*, *d*, and *z*. Further, a Bonferroni-corrected threshold of *P* < 0.10 was applied to control for multiple testing. All statistical analyses were done using the software R (R Development Core Team 2014) and the package ASReml-R 3.0 (Gilmour et al. 2009). The minor allele frequency (MAF) for significant associated markers was calculated and the linkage disequilibrium (LD) was assessed by the LD measure *r*^2^ (Weir 1996).

## Results

**Fig. 1:**
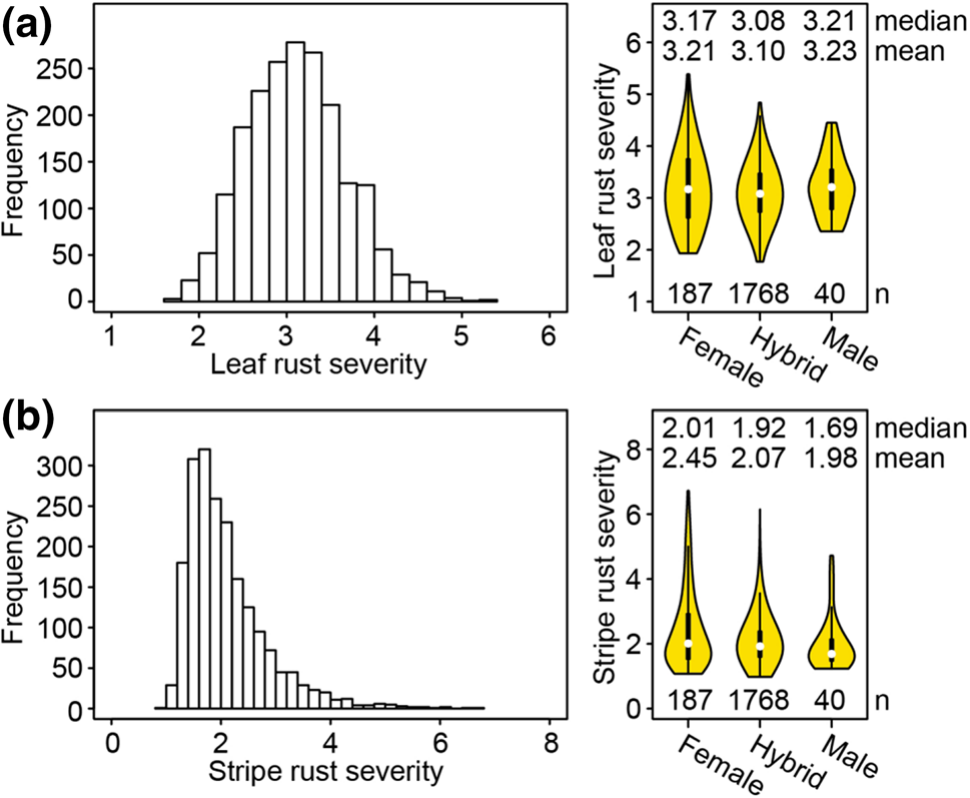
Summary of phenotypic data for leaf rust (**a**) and stripe rust severity (**b**). Histograms showing genotype frequencies for scoring grades one to nine on the x-axis for leaf rust and stripe rust severity. In addition, violin plots showing the distribution for rust severity clustered for single parental pools and hybrids

The hybrids and their parental lines were evaluated for leaf rust severity in seven different environments. The estimated heritability for parents was *h*^2^ = 0.82 and for hybrids *h*^2^ = 0.66. The sum of variance of GCA effects was two times larger than the variance of SCA effects (Suppl. Table 1). Leaf rust severity of female parents ranged from 2 to 5 with a mean of 3.21 and male lines ranged from 3 to 4 and averaged at 3.23 (Fig. 1; Suppl. Table 2). The hybrid population covered a range from 2 to 5 with a mean value of 3.10. The hybrids outperformed the better parent by an average of 0.28 (Suppl. Figure 1; Suppl. Table 2).

The assessment of different genotypes for stripe rust severity was conducted in five environments resulting in a heritability for parents and hybrids of *h*^2^ = 0.92 and *h*^2^ = 0.72, respectively. The sum of variance of GCA effects was five times larger than the variance of SCA effects (Suppl. Table 1). The parental pool of females showed a wide distribution from 1 to 7 with an average of 3.45, while male lines ranged from 1 to 5 with a mean of 1.98. Hybrid scores were ranging from 1 to 6 with a mean of 2.07 (Fig. 1; Suppl. Table 2). The stripe rust distribution was skewed towards resistance, which can be explained by intensive selection of parental lines for this trait. The hybrids outperformed the better parent by an average of 0.40 (Suppl. Figure 1; Suppl. Table 2).

Parental lines were genotyped with genome-wide distributed SNP markers. We examined the population structure and relatedness of all parental lines by calculating their Rogers’ distances (Suppl. Figure 2) and implemented a PCoA (Suppl. Figure 3). Both procedures indicated that the population of parental lines is not structured and distinct parental pools are missing. Therefore, we corrected in the genome-wide association mapping for relatedness using a kinship matrix and ignored terms to correct for effects of subpopulations.

**Fig. 2:**
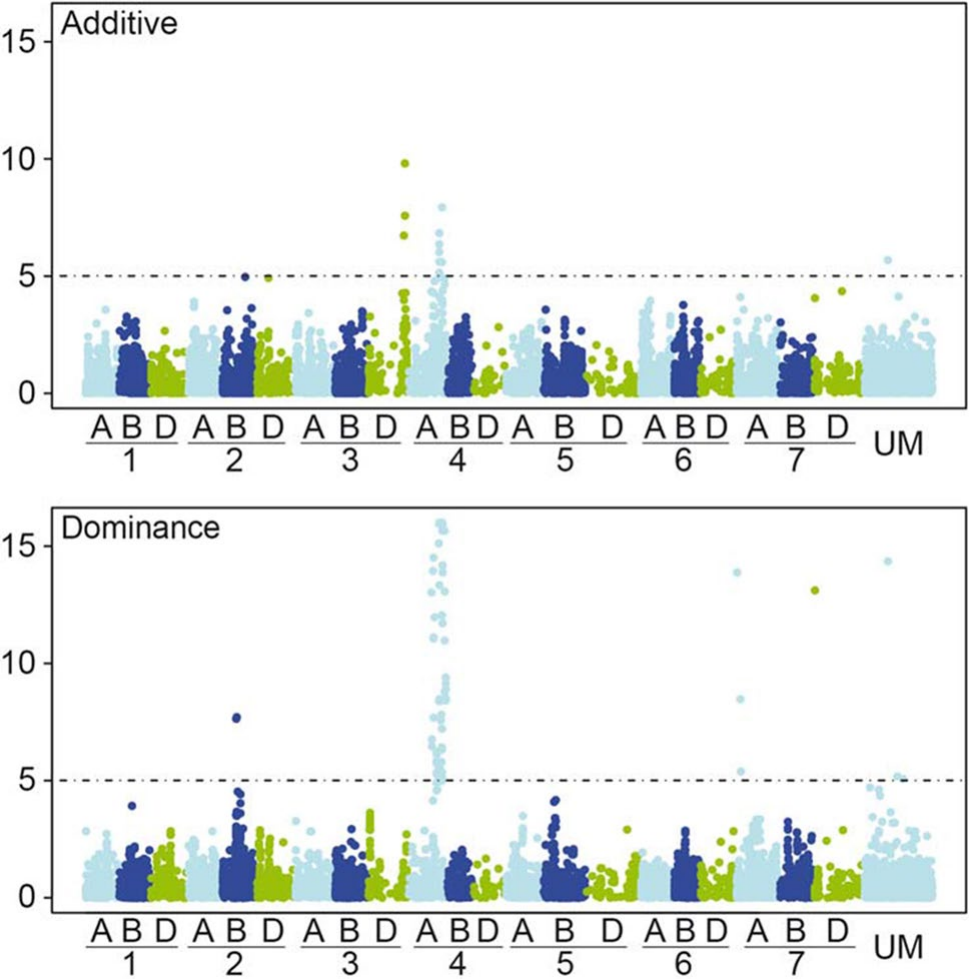
Manhattan plots from the genome-wide association scan for additive and dominance effects on leaf rust severity. The dashed horizontal line symbolizes the significant threshold of *P* < 0.10 applying Bonferroni correction. Unmapped markers were outlined under “UM”

Genome-wide association scans were performed with the high-quality data of 10,453 markers using a significance threshold of *P* < 0.10 applying Bonferroni correction for multiple testing. We observed only a few loci with significant additive effects on leaf rust severity. In contrast, there were several loci exhibiting significant dominance effects (Fig. 2). Putative quantitative trait loci (QTL) affecting leaf rust resistance were detected on chromosomes 3D and 4A with respect to their additive effects and on chromosomes 2B, 4A, and 7A for dominance effects. The strongest association for leaf rust resistance was identified for dominance effects by a major peak of significantly associated markers on chromosome 4A. In total, there were 61 significantly associated markers on chromosome 4A, which were all located between 104 and 166 cM according to the genetic linkage map of Wang et al. (2014). Those associated markers could be assigned to 22 or 47 unique groups based on similar genetic or physical positions. Most of the identified markers with an effect on leaf rust resistance are physically located on chromosome 4A in the genomic region between 627,815 and 742,567 Mbp of the wheat reference genome (International Wheat Genome Sequencing Consortium (IWGSC) 2018). Combining the marker data with the wheat reference genome was done to compare associated loci with genomic regions of previous known function. Four detected significant markers on chromosome 4A located between 707,043 and 726,215 Mbp in addition to two markers on 2B placed at 157,694 and 442,797 Mbp were identified within the regions of previous known NBS-LRR genes, while detected markers on chromosome 2B and 6A are located within regions of previous known protein kinase genes (Table 2). Most of the identified significant markers showed desired negative dominance effects (Suppl. Table 3). Based on the implemented analyses, putative QTLs were identified on chromosomes 2B, 3D, 4A, and 7A.

**Table 2:** Comparison of detected markers with a significant effect on leaf rust severity with the location of previously known resistance genes within the reference genome

Marker (ID of reference gene)	Type	Chr.	Physical Marker Pos.		Gene function	Physical Gene Pos.	
			Start (bp)	End (bp)		Start (bp)	End (bp)
RAC875_c1226_652 (TraesCS2B01G182800)	dom	2B	157,693,634	157,693,534	NBS-LRR disease resistance protein	157,688,966	157,696,282
wsnp_JD_c9251_10121369 (TraesCS2B01G309900)	dom	2B	442,796,772	442,796,972	Protein kinase	442,795,590	442,798,055
wsnp_Ex_c4331_7808746 (TraesCS4A01G437200)	add	4A	707,042,951	707,043,104	Protein enhanced disease resistance 2-like	707,040,590	707,048,030
Excalibur_rep_c112888_602 (TraesCS4A01G446700)	add	4A	714,176,967	714,176,867	Disease resistance protein (TIR-NBS-LRR class) family	714,176,254	714,180,521
RAC875_rep_c69632_65 (TraesCS4A01G446700)	add	4A	714,179,046	714,179,146	Disease resistance protein (TIR-NBS-LRR class) family	714,176,254	714,180,521
BobWhite_c47168_598 (TraesCS4A01G461700)	add	4A	726,214,991	726,214,891	NBS-LRR resistance-like protein	726,212,910	726,217,457
BobWhite_c47168_289 (TraesCS4A01G461700)	add	4A	726,215,300	726,215,200	NBS-LRR resistance-like protein	726,212,910	726,217,457
wsnp_RFL_Contig4456_5258284 (TraesCS6A01G401200)	add	6A	609,820,815	609,820,915	Leucine-rich repeat receptor-like protein kinase family protein	609,813,988	609,821,699

**Fig. 3:**
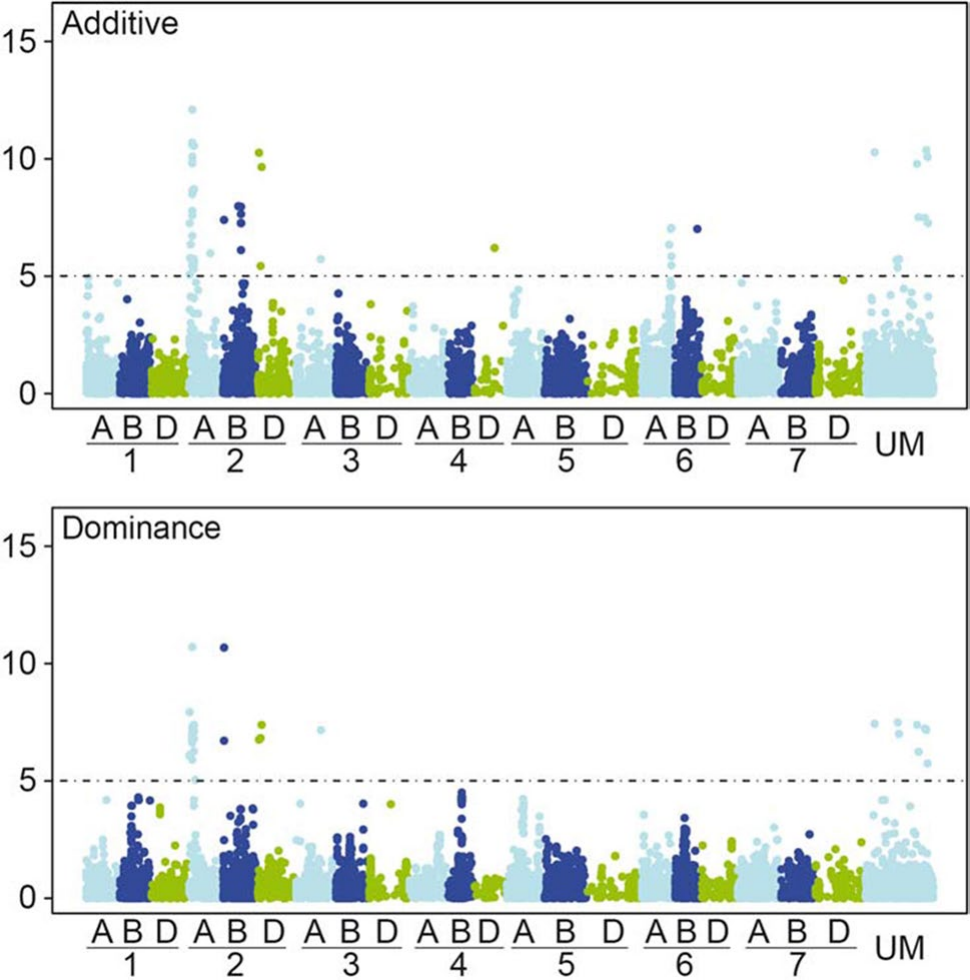
Manhattan plots of the genome-wide association scan for additive and dominance effects on stripe rust severity. The dashed horizontal line symbolizes the significant threshold of *P* < 0.10 applying Bonferroni correction

Several loci with significant additive effects, but only a few with significant dominance effects on stripe rust severity were detected (Fig. 3). Strong associations with major peaks of significantly associated markers were identified on chromosomes 2A and 2B for both, additive and dominance effects and on chromosome 6A for additive effects. We detected 25 significantly associated markers on chromosome 2A, which are located between 2.9 and 29.1 cM corresponding to the genetic linkage map of Wang et al. (2014). The majority of identified markers on chromosome 2A, which were associated with stripe rust resistance were physically located in the genomic region between 2478 and 19,126 Mbp of the wheat reference genome (IWGSC 2018). The referred physical regions were used to identify significant associated markers related to genome regions with reported resistance functions. A few detected significant markers were located in the regions of previously known NBS-LRR genes on chromosome 2A (Table 3) and may be considered as possible candidate genes for different *Yr*-genes. Most of the identified significant markers showed desired negative dominance effects (Suppl. Table 4).

**Table 3:** Comparison of detected markers with a significant effect on stripe rust severity with the location of previously known resistance genes within the reference genome

Marker (ID of reference gene)	Type	Chr.	Physical Marker Pos.		Gene function	Physical Gene Pos.	
			Start (bp)	End (bp)		Start (bp)	End (bp)
BS00068050_51 (TraesCS2A01G016200)	add	2A	7,514,010	7,514,110	NB-ARC domain-containing disease resistance protein	7,513,301	7,514,256
BobWhite_c12426_84 (TraesCS2A01G030000)	add/dom	2A	13,814,670	13,814,570	NBS-LRR-like resistance protein	13,811,892	13,815,254
CAP12_c259_307 (TraesCS2A01G037200)	dom	2A	15,875,831	15,875,893	NBS-LRR resistance-like protein	15,874,900	15,876,274
D_contig01272_220 (TraesCS2A01G053200)	add/dom	2A	21,262,837	21,263,050	NBS-LRR disease resistance protein	21,261,141	21,264,604

**Fig. 4:**
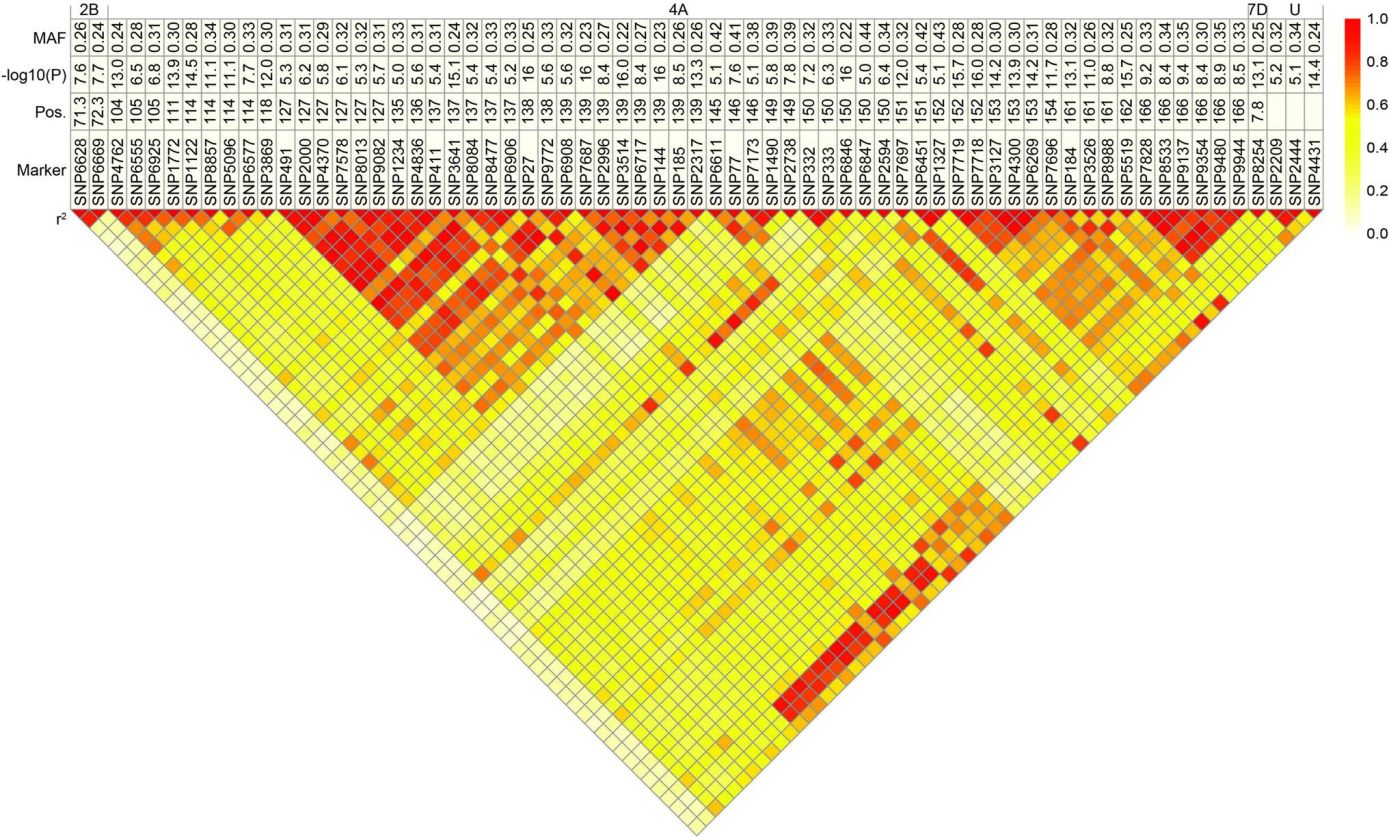
SNPs with dominant effects for leaf rust severity. Table including minor allele frequency (MAF), significance value (−log 10(P)), and genetic map position of respective SNP markers that contribute significantly to the dominant genetic variation of leaf rust severity. The heat plot presents the linkage disequilibrium (LD) measured as squared Pearson’s correlation coefficients (*r*^2^) among SNP markers

**Fig. 5:**
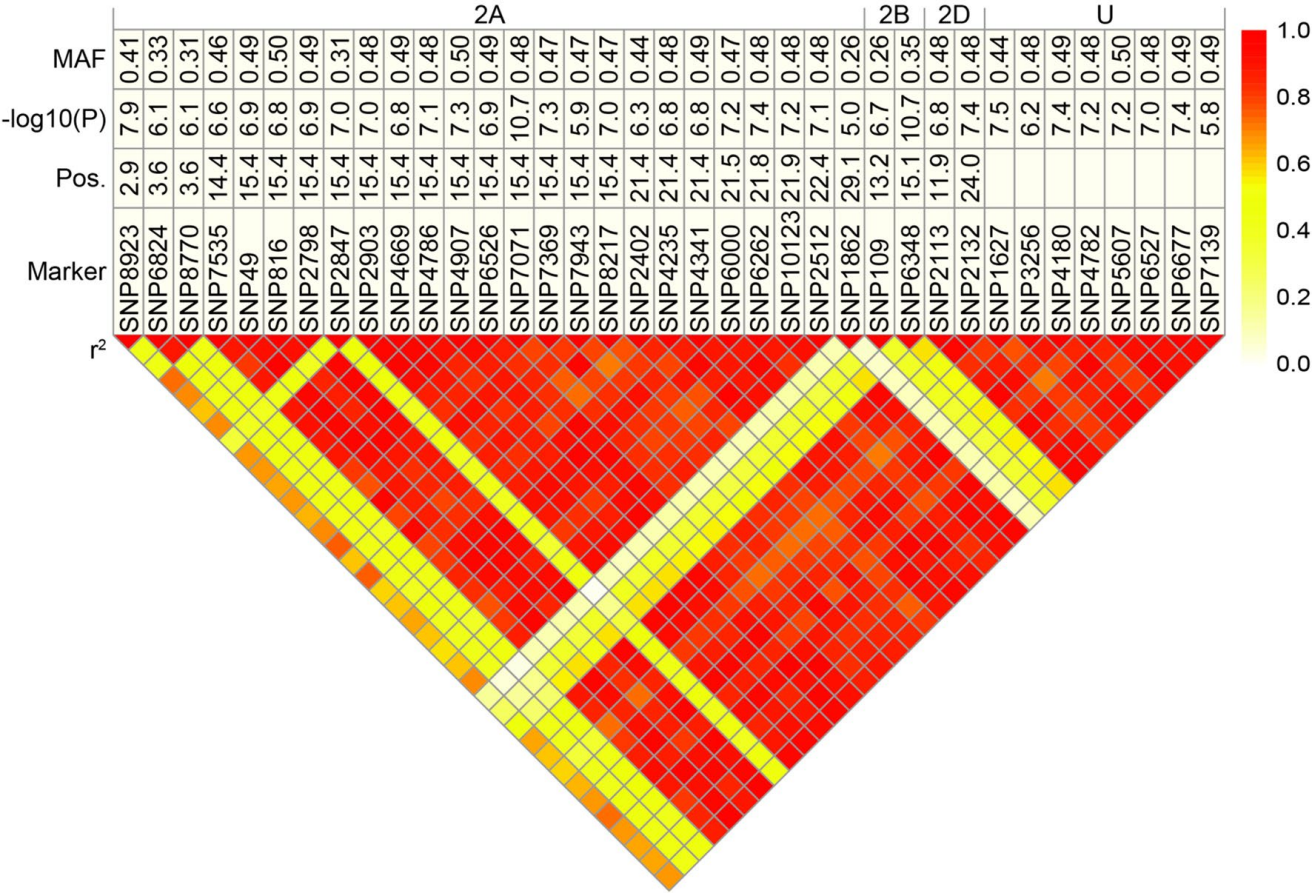
SNPs with dominance effects for stripe rust severity. Table including minor allele frequency (MAF), significance value (−log 10(P)), and genetic map position of respective SNP markers that contribute significantly to the dominant genetic variation of stripe rust severity. The heat plot presents the linkage disequilibrium (LD) measured as squared Pearson’s correlation coefficients (*r*^2^) among SNP markers

**Fig. 6:**
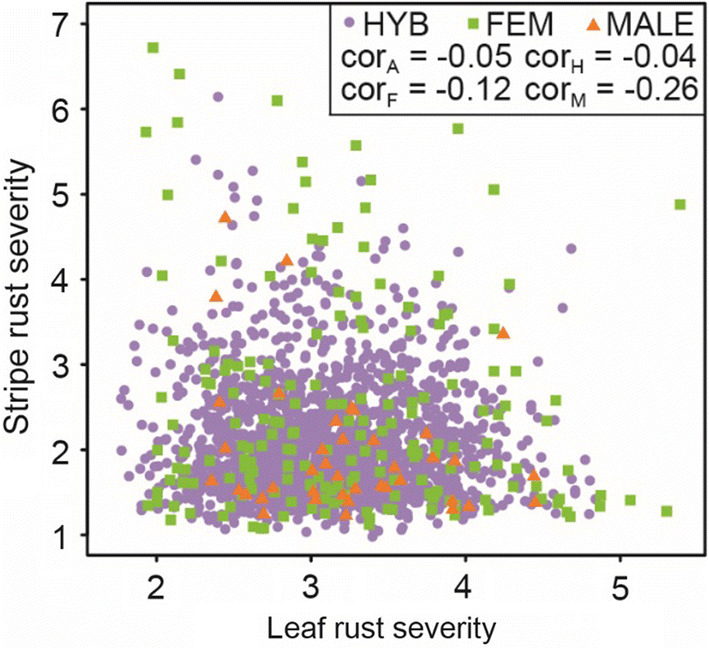
Plot of BLUEs for each phenotyped genotype comprising parents and hybrids. The x-axis represents the score for leaf rust severity while on the y-axis the stripe rust severity is shown. Furthermore, the correlation between leaf rust and stripe rust severity for parental groups, hybrids, and the whole population is presented

**Fig. 7:**
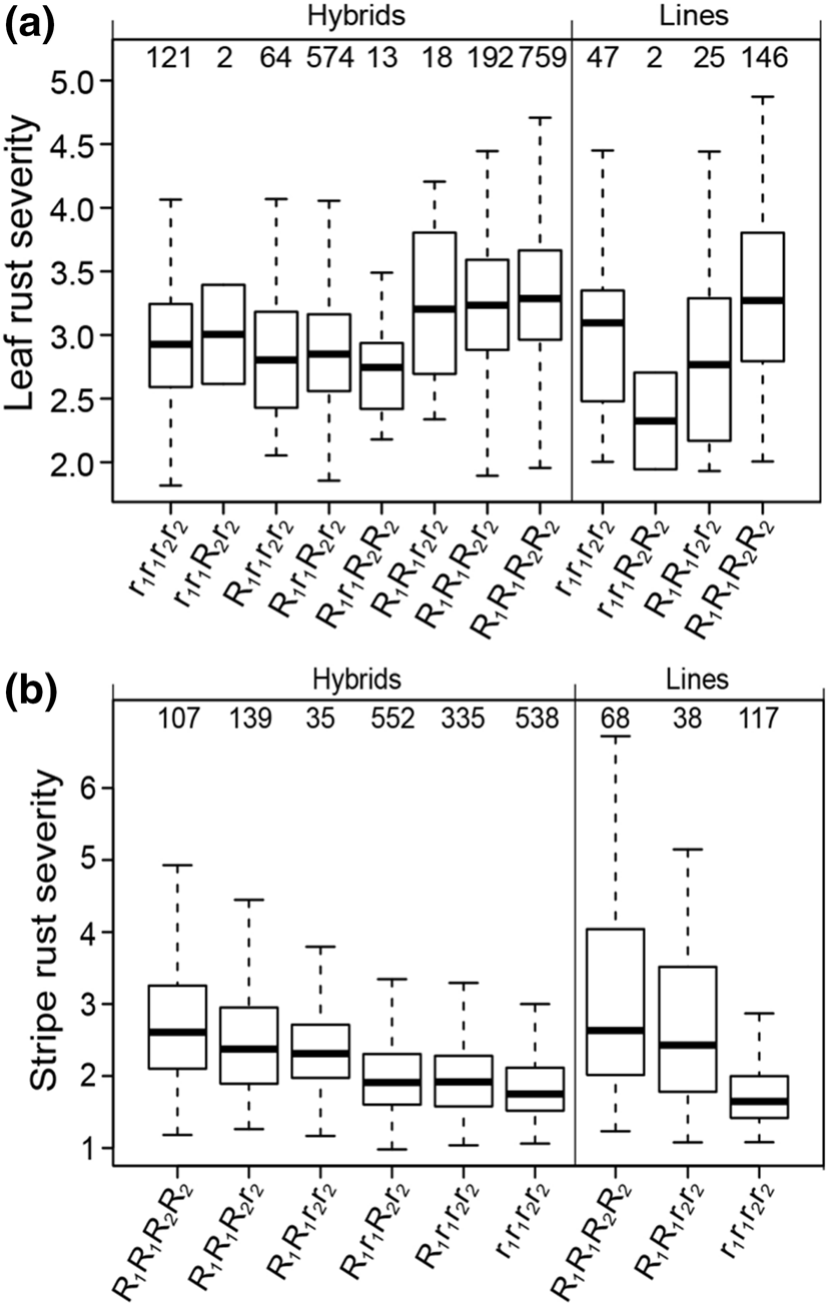
Leaf rust (**a**) and stripe rust resistance (**b**) in dependence on genotypes detected by associated SNP markers. Box-whisker plots showing leaf rust severity of adult plants for different allele combinations at two resistance gene loci explaining each ≥ 12% of the phenotypic variation. SNP6846 (*r*_1_/*R*_1_) and SNP6577 (*r*_2_/*R*_2_) as well as SNP7071 (*r*_1_/*R*_1_) and SNP8770 (*r*_2_/*R*_2_) were observed for leaf and stripe rust resistance, respectively. *R* refers to the allele supporting susceptibility, while *r* represents the allele increasing resistance. The numbers at the top of each box refer to the observed numbers of hybrids (left) and parental inbred lines (right). Only homozygous parental lines and hybrids derived from them were considered

By the analysis of the linkage disequilibrium (LD), patterns among significantly associated markers were determined using the squared Pearson´s correlation coefficient (*r*^2^) among the SNP markers (Figs. 4, 5; Suppl. Figures 4, 5). The markers can be clustered into two groups, while there is a high LD within each group and moderate LD between the groups. Comparing the phenotypic information for the two rust diseases unraveled an absence of a correlation between leaf rust and stripe rust severity (Fig. 6). Further on, the few markers explaining the highest phenotypic variance for leaf rust and stripe rust resistance were used to examine the dominance degree (Fig. 7). The degree of dominance is high ranging for the observed markers from partial dominance to overdominance.

## Discussion

During the last decade, intensive research and development efforts have been spent to establish hybrid breeding programs for self-pollinating species. In comparison to their parental inbred lines, wheat hybrids showed on average around 10% of grain yield increase (Longin et al. 2013) and a higher yield stability (Mühleisen et al. 2014). Furthermore, wheat hybrids were on average less susceptible against biotic and abiotic stresses (Gowda et al. 2014; Longin et al. 2013; Miedaner et al. 2013; Zhao et al. 2013) and allow to stack major dominant genes during the breeding process (Longin et al. 2012). Leaf rust and stripe rust belong to the most important fungal diseases of wheat (Huerta-Espino et al. 2011; Wellings 2011). Due to a continuous development of naturally occurring rust populations, epidemics appear in high frequency and can cause yield losses of up to 70% (Chen 2005; Huerta-Espino et al. 2011) as well as a reduction in grain quality (Prescott et al. 1986). In this study, we explored the resistance of hybrids against leaf rust and stripe rust in comparison to their parental inbred lines. Phenotypic data were collected from 1750 hybrids and 230 parental lines and were combined with genotypic marker information to elucidate the genetic architecture by performing genome-wide association mapping.

### Intensive phenotyping resulted in precise estimates of leaf rust and stripe rust severity

Phenotyping for leaf rust and stripe rust resistance was done under natural infection conditions in field trials across seven and five environments, respectively. The present conditions led to natural infections with leaf rust and stripe rust within each field trial. The quality of field trials was evaluated by analyzing single locations and studying the genomic heritability within each location. The minimum value observed for genomic heritability amounted to 0.37 with a mean of 0.57. We consider this as an indicator for excellent testing conditions. For the comparison with other studies, the plot-based heritability for hybrids was estimated and showed values of 0.22 and 0.34 for leaf rust and stripe rust, respectively. Longin et al. (2013) observed comparable values for resistance against stripe rust (0.25), powdery mildew (0.49), and septoria tritici blotch (0.29). This accordance confirms the high quality of the collected field data within this study.

### Specific hybrid combinations outperformed parental lines for leaf rust and stripe rust resistance

The resistance level of male and female lines was comparable for leaf rust (Fig. 1; Suppl. Table 2). In contrast, females were more susceptible than males for stripe rust. For the female pool, $\sigma_{\text{GCA}}^{2}$ contributes to 16.5% of the total genetic variance for stripe rust, which illustrates the potential to improve the resistance within the female pool. Females showed a higher phenotypic diversity than male parents, which was reflected by four and two times larger $\sigma_{\text{GCA}}^{2}$ and the variance of phenotypic resistance performance against leaf rust and stripe rust, respectively.

Average midparent heterosis (MPH) amounted to − 1.6% and − 1.7% for leaf rust and stripe rust resistance, respectively. Better parent heterosis (BPH) was on average 12% and 26% for leaf rust and stripe rust resistance, respectively. In total 31% (556 genotypes) and 22% (399 genotypes) of the hybrids are more resistant than their better parent with a maximum of − 56% (improvement of 2.5 scores) and − 47% (improvement of 1.6 scores) for leaf rust and stripe rust resistance, respectively. Those results were in accordance with previous findings of Longin et al. (2013), who observed comparable amounts of average midparent heterosis for leaf rust and stripe rust resistance. In summary, hybrid heterosis for leaf rust and stripe rust resistance cannot be taken as generally valid concept. Specific hybrid combinations showing valuable heterosis effects for rust resistance are required to exploit the potential of hybrid wheat breeding. The large variation in heterosis levels leads to the conclusion that a clear understanding of the genetic architecture influencing the hybrid performance is essential to use the portfolio of resistance genes in an optimal way for hybrid breeding.

### Loci on 3D, 4A and 2A, 2B, 6A were strongly associated to leaf rust and stripe rust resistance, respectively

Performing genome-wide association mapping with regard to leaf rust resistance revealed significantly associated loci on chromosomes 3D and 4A. The identified markers explained each a phenotypic variance ranging between 3–4% (4.45% for the marker with the highest *P* value), 9–12% (12.3% for the marker with the highest *P* value), and 5–12% (12.31% for the marker with the highest *P* value) (Suppl. Table 3). Chromosome 4A appears to be an important genome region with a high potential for leaf rust resistance in wheat. Correspondingly, promising loci were previously detected in association studies observing adult plant resistance in a set of American spring wheat (Gao et al. 2016) and in a diverse core set including winter and spring wheat (Li et al. 2016) or considering seedling resistance in a wheat collection of CIMMYT (Juliana et al. 2018). The detected single nucleotide polymorphism (SNP) *BobWhite_c47168_289* (SNP332) was also found as significantly associated with leaf rust resistance on wheat chromosome 4A by Gao et al. (2016). Significantly associated marker on chromosome 3D did not match with previous known gene functions connected to disease resistance (IWGSC 2018). Previous detected *Lr*-genes on chromosome 3D did not occur in the European wheat population. Detected loci from this study are located inside genome regions of previous known nucleotide-binding site leucine-rich repeat (NBS-LRR) genes (IWGSC 2018). It would be obvious to treat the detected loci as candidate genes for the known leaf rust resistance genes *Lr28* and *Lr30*, which were located on chromosome 4AL (McIntosh et al. 2013) while *Lr28* is more likely because of its known dominant inheritance (McIntosh et al. 1982) in contrast to *Lr30* (Kolmer 1996). Further work needs to be conducted for verification, because those resistance genes were not cloned so far and due to the large size of chromosome 4AL (~ 300 Mb) NBS-LRR genes might occur in a high frequency (IWGSC 2018). Loci showing a strong association with stripe rust resistance were detected on chromosomes 2A, 2B, and 6A. However, identified markers on chromosome 2B and 6A did not match with previous known resistance genes (IWGSC 2018). There is no previous identified *Yr*-gene on chromosome 6A. In contrast, marker-trait associations on chromosome 2B are putative candidates for *Yr27* (National BioResource Project 2018), which occur with a high frequency in the European wheat population. These SNPs explained 3–16% (15.83% for the marker with the highest *P* value) of the phenotypic variance for markers on chromosome 2A, 5–11% (10.92% for the marker with the highest *P* value) of the phenotypic variance for markers on chromosome 2B, and 1–3% (2.74% for the marker with the highest *P* value) of the phenotypic variance for markers on chromosome 6A, respectively (Suppl. Table 4). The importance of chromosome 2A for seedling and adult plant resistance in wheat was also shown by Juliana et al. (2018) considering CIMMYT´s bread wheat pool. Positions of detected loci on 2A are in coincidence with previous detected NBS-LRR genes (IWGSC 2018). It is known that seven different stripe rust resistance genes are located on chromosome 2A (*Yr1*, *Yr17*, *Yr32*, *Yr61*, *Yr56*, *Yr69*, *YrJ22*) (National BioResource Project 2018). The European wheat population is segregating for the resistance genes *Yr1*, *Yr17*, and *Yr32*, which are present at a high frequency especially in winter wheat varieties originating from France and Great Britain (National BioResource Project 2018). *Yr1* and *Yr32* were reported to be located on the long chromosome arm of 2A (Eriksen et al. 2004; National BioResource Project 2018), while in contrast, *Yr17* originally translocated from *Aegilops ventricosa* could be identified on 2AS (Seah et al. 2001). The wheat chromosome 2A has a total size of 780.8 Mb (IWGSC 2018) and the identified loci related to a resistance function were located ranging from 7.5 to 21.2 Mb (Table 3). On this account, it is obvious to assume that the few examined loci are located on the short chromosome arm of 2A and were therefore most likely treated as candidate genes for *Yr17.*

### Leaf rust and stripe rust severity is not correlated in the analyzed European breeding material

The comparison of genotypic values for leaf rust and stripe rust severity revealed no association between both traits. This is in contrast to previous findings, which reported the presence of a few pleiotropic genes in wheat causing resistance for both leaf rust and stripe rust. Published completely linked genes are *Lr34*/*Yr18* (McIntosh 1992), *Lr46*/*Yr29* (William et al. 2003), *Lr27*/*Yr30* (Singh 1992), and *Lr67*/*Yr46* (Herrera-Foessel et al. 2011) located on chromosome 7DS, 1BL, 3BS, and 4DL, respectively. Those pleiotropic genes are predominantly present in spring wheat varieties of North and South America (National BioResource Project 2018) and were identified in this study, therefore. However, *Lr34*/*Yr18* and *Lr27*/*Yr30* have been implemented in many wheat cultivars, but *Lr34* causes extremely small quantitative resistance effects under field conditions, which are macroscopically hardly visible. The resistance induced by *Lr27* was broken down by many isolates of the natural occurring leaf rust populations (Dadkhodaie et al. 2011) and was therefore not detected. This leads to the assumption that pleiotropic genes causing resistance against both rust diseases are absent or inefficient in European winter wheat varieties and explains the lack of an association between leaf rust and stripe rust resistance in our study.

### Improved resistances can be achieved through hybrid breeding by fixing leaf rust and stripe rust resistance genes in opposite parental pools

To reach an increased resistance level of hybrids with the help of detected recessive resistance genes, it would be necessary to fix those genes in both parental pools. In this case improving resistance is not simplified by hybrid compared to line breeding. In contrast, 72 and 56 SNPs with significant dominance effects were identified overall influencing leaf rust and stripe rust resistance, respectively. The majority of those markers (60% for leaf rust and 98% for stripe rust) showed higher absolute additive than absolute dominance effects. The dominance effect was negative for all detected markers, leading to a decreased rust susceptibility in the hybrids compared to the midparent performance. Based on the amount and the relation of additive and dominance effects, most marker loci may be assumed as partial dominant for both, leaf rust and stripe rust resistance. In correspondence to that, Gowda et al. (2014) detected partial dominance for leaf rust and stripe rust resistance studying a hybrid winter wheat set. Despite of the frequent occurrence of partial dominance, a fixation of resistance genes in only one parental pool may beneficial. The partial dominance pushing the resistance to a higher level. Therefore, fixing the favorable resistance allele in one parental pool would minimize breeding efforts and save resources, while resulting in a gain of rust resistance (Fig. 7). A rust resistance based on various loci would maximize the resistance benefit for heterozygous hybrids and would have additionally a positive effect on the effectiveness as well as the durability of the resistance against fast adapting rust populations. Furthermore, 8% of the examined marker loci showed a complete dominance for leaf rust resistance, while 32% and 2% of the loci represented overdominance for leaf and stripe rust resistance, respectively (Suppl. Tables 3, 4). For those marker loci, the dominant resistance gene should be fixed in only one parental pool for the production of resistant hybrids. This would simplify the accumulation of different resistance genes within a hybrid due to the combination of parental inbred lines carrying different resistance loci (Longin et al. 2013) and is therefore a big advantage of the hybrid breeding strategy. In summary, the requirement for a desirable and profitable fixation of resistance genes in only one parental pool is the existence of specific hybrid combinations showing favorable partial or complete dominance for disease resistance, and those genes are needed to exploit the potential of hybrid wheat breeding. To fulfill this and identify favorable hybrids, a detailed knowledge of the degree of dominance for the relevant resistance genes is needed.

## Electronic supplementary material

Below is the link to the electronic supplementary material.

Supplementary material 1 (DOCX 4505 kb)
