# Supplementary material for: The potential of hybrid breeding to enhance leaf rust and stripe rust resistance in wheat
Source: Theor Appl Genet. 2020 Apr 12;133(7):2171–81. doi: 10.1007/s00122-020-03588-y (PMC7311497; doi:10.1007/s00122-020-03588-y)

**Supplementary Information**

**Suppl. Table 1:** Table of summarizing statistics showing results of variance components analysis.

|  | **Leaf rust** | **Stripe rust** |
| --- | --- | --- |
| No. of environments | 7 | 5 |
| $\sigma_{Env}^{2}$ | 1.32 | 0.21 |
| $\sigma_{Block}^{2}$ | 0.07 | 0.15 |
| $\sigma_{error}^{2}$ | 1.26 | 0.91 |
| Parental lines |  |  |
| $\sigma_{Genotype}^{2}$ | 0.82 | 2.05 |
| $h^{2}$ | 0.82 | 0.92 |
| Hybrids |  |  |
| $\sigma_{GCA female}^{2}$ | 0.22 | 0.34 |
| $\sigma_{GCA male}^{2}$ | 0.05 | 0.17 |
| $\sigma_{SCA}^{2}$ | 0.13 | 0.11 |
| $\sigma_{GCA female x Env}^{2}$ | 0.14 | 0.17 |
| $\sigma_{GCA male x Env}^{2}$ | 0.06 | 0.11 |
| $h^{2}$ | 0.66 | 0.72 |

**Suppl. Table 2:** Descriptive statistics summarizing phenotypic data of hybrids and parental lines observing leaf rust and stripe rust severity.

|  | **Leaf rust severity** | **Stripe rust severity** |
| --- | --- | --- |
| Female lines |  |  |
| min | 1.93 | 1.08 |
| max | 5.39 | 6.72 |
| mean | 3.23 | 2.43 |
| Male lines |  |  |
| min | 2.36 | 1.23 |
| max | 4.45 | 4.72 |
| mean | 3.24 | 1.98 |
| Hybrids |  |  |
| min | 1.77 | 0.98 |
| max | 4.84 | 6.15 |
| mean | 3.11 | 2.08 |
| min MPH | -2.68 | -2.42 |
| max MPH | 1.81 | 2.65 |
| mean MPH | -0.08 | -0.10 |
| min BPH | -2.47 | -1.61 |
| max BPH | 2.31 | 4.60 |
| mean BPH | 0.28 | 0.4 |

**Suppl. Figure 1:** Boxplot showing the distribution of mid-parent (MPH) and better parent heterosis (BPH) for leaf rust and stripe rust severity based on a phenotyping scheme of nine classes, where 9 is fully susceptible and class 1 is resistant.


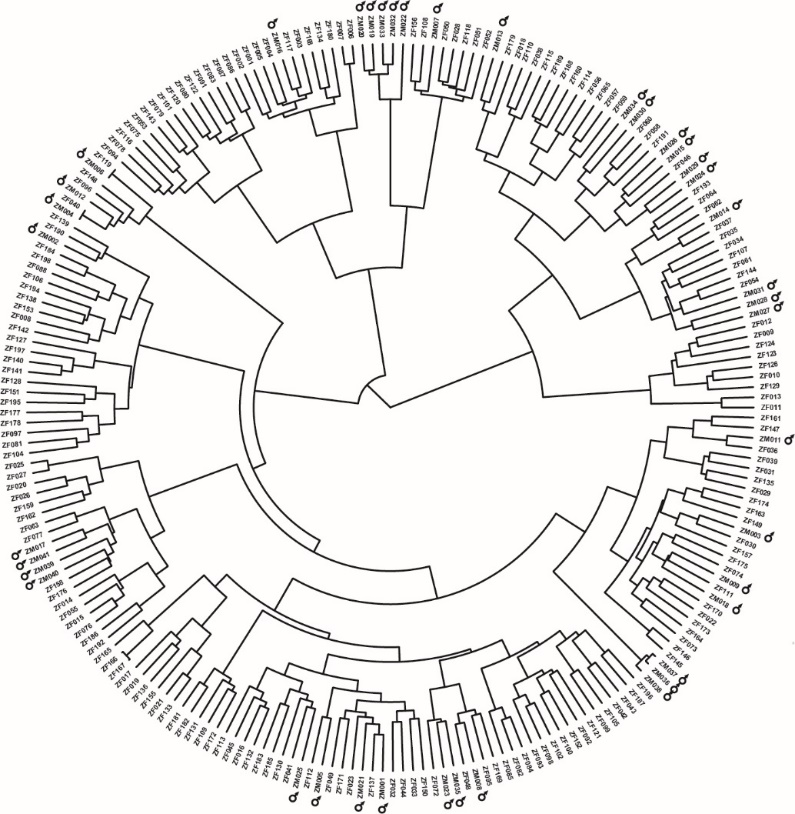


**Suppl. Figure 2:** Population structure of parental lines. Tree plot showing relationship of parental lines based on their calculated Rogers’ distances. Joining lines representing relatedness between genotypes. The more junctions there are the wider is the relation between two individuals. The 41 male parents are highlighted by male gender symbols.


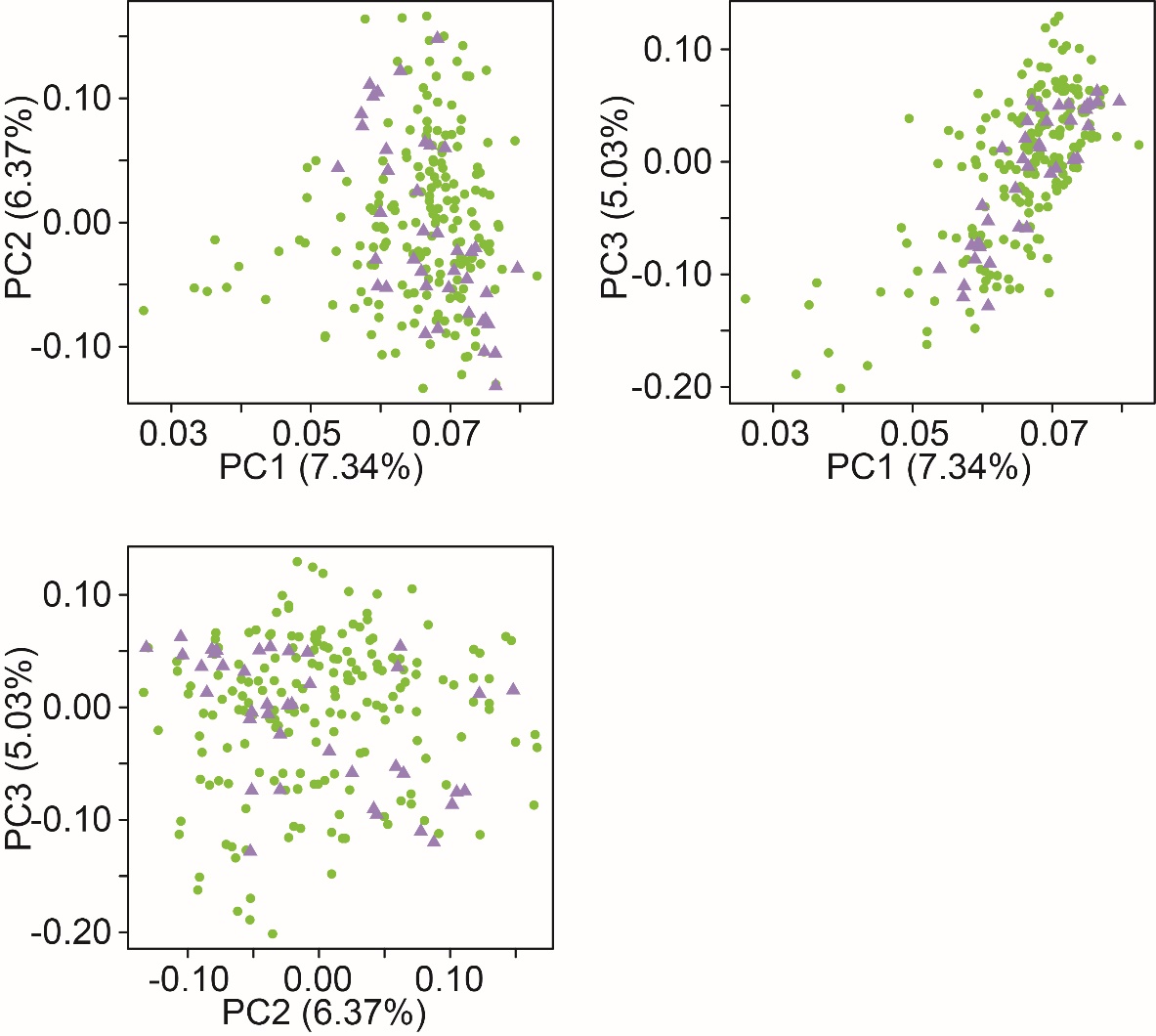


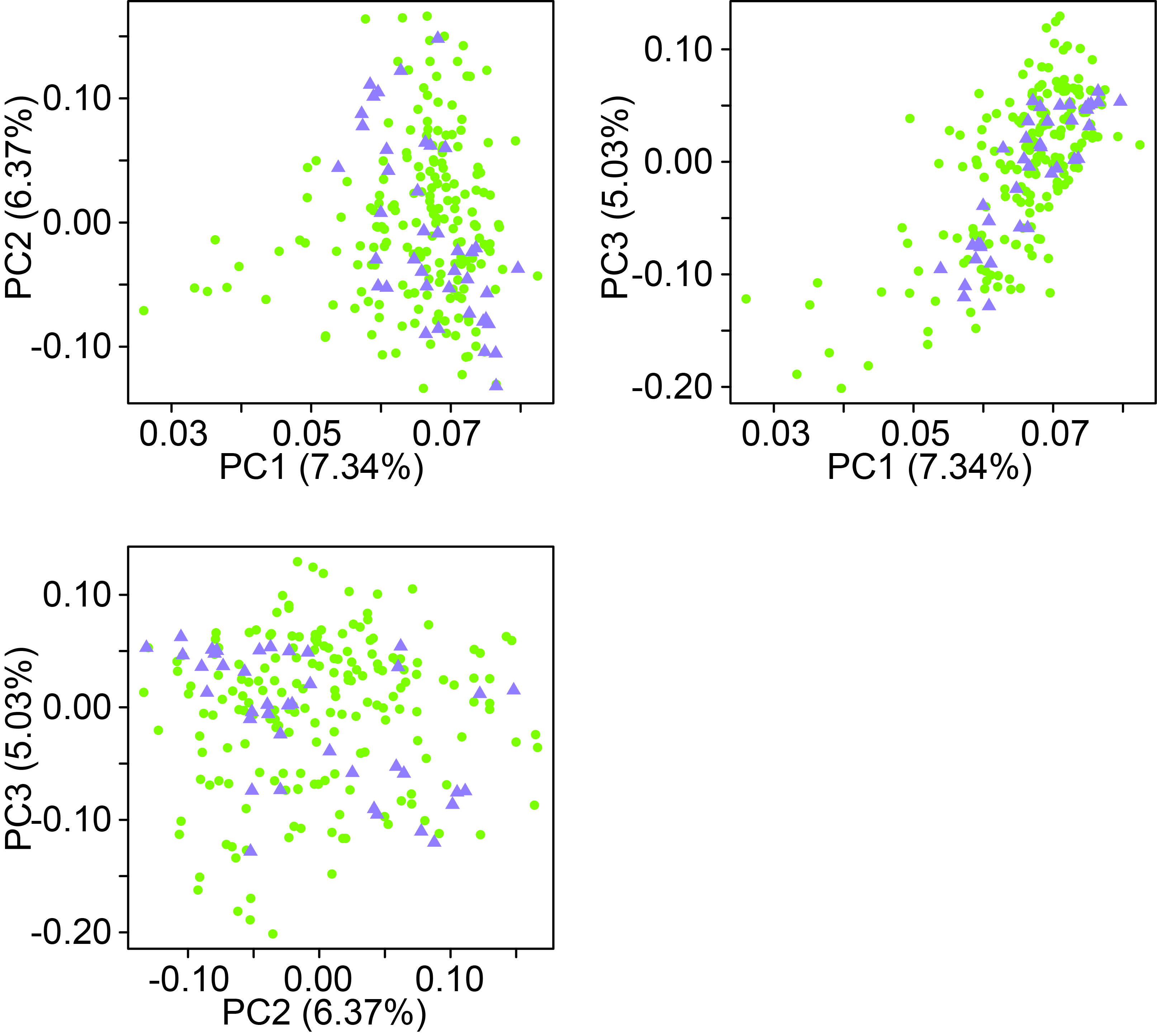
 Male lines


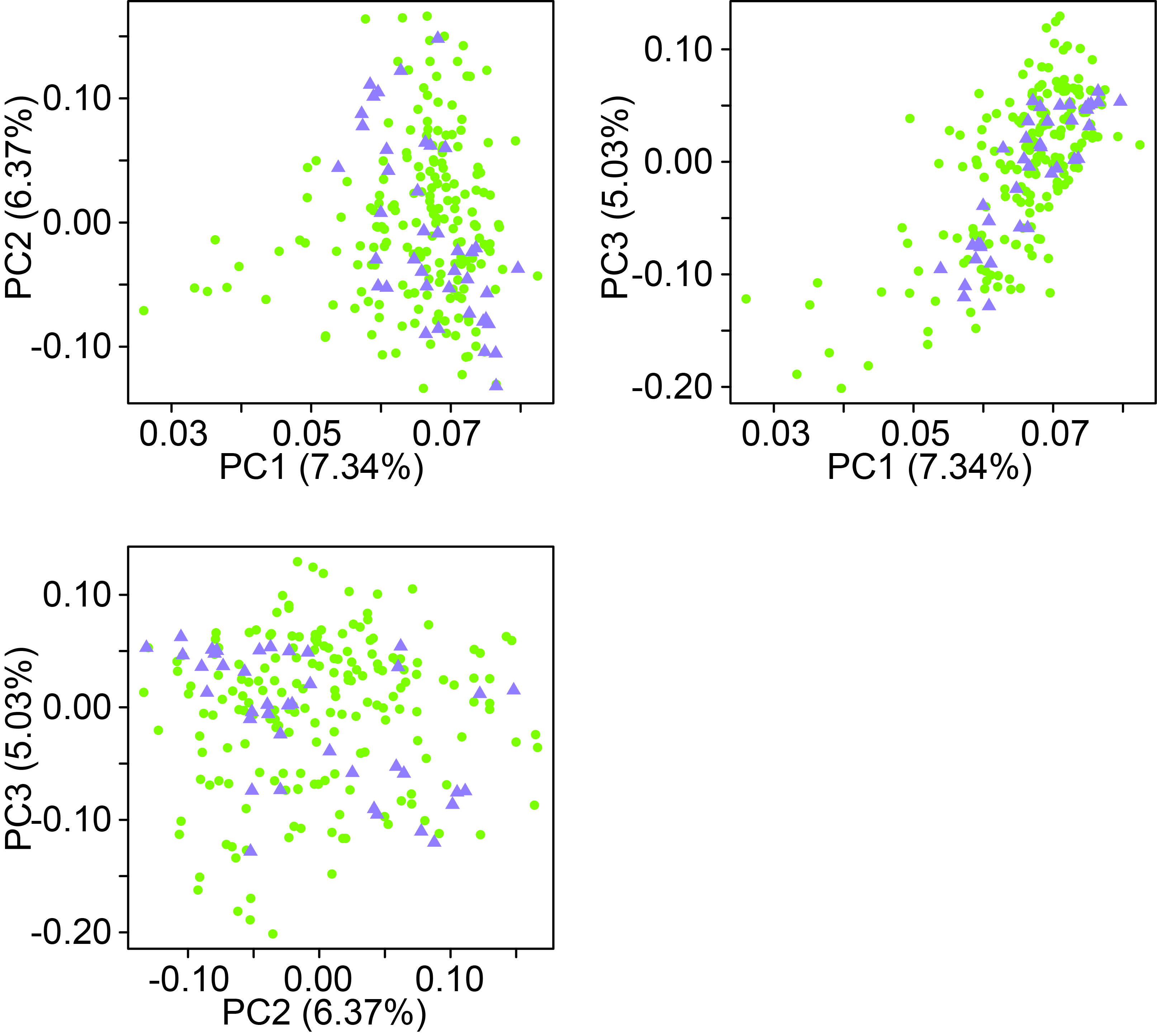
 Female lines

**Suppl. Figure 3:** Population structure of parents. Plot of principal coordinate analysis observing the first three coordinates. Proportions of genotypic variance explained by the principal coordinates are indicated in brackets. Lilac triangles symbolize male lines and green dots female lines.

**Suppl. Table 3:** Loci significantly associated with leaf rust resistance, their chromosomal (Chrom.) position (Pos.), the additive (Add.) and dominance (Dom.) effects, and the adjusted (Adj.) R².

| **Marker (SNP)** | **Type** | **Chrom.** | **Pos. (cM)** | **Add. effect** | **Dom. effect** | **Adj. R^2^ (%)** |
| --- | --- | --- | --- | --- | --- | --- |
| RAC875_rep_c106400_276  (SNP6628) | add | 2B | 71.261 | 0.09 | -0.16 | 4.05 |
| RAC875_rep_c112008_519  (SNP6669) | add | 2B | 72.335 | 0.08 | -0.17 | 4.25 |
| Kukri_c4230_398  (SNP5030) | dom | 3D | 170.388 | -0.18 | -0.09 | 2.94 |
| Ra_c72650_1176  (SNP5672) | dom | 3D | 175.389 | -0.18 | -0.1 | 3.13 |
| Ra_c6639_426  (SNP5656) | dom | 3D | 175.389 | 0.18 | -0.15 | 4.45 |
| RFL_Contig2531_987  (SNP6847) | add | 4A | 149.752 | 0.14 | -0.12 | 4.85 |
| BS00065030_51  (SNP1490) | add | 4A | 148.658 | 0.15 | -0.11 | 5.53 |
| Excalibur_c13811_1086  (SNP2738) | add | 4A | 148.658 | 0.15 | -0.13 | 6.07 |
| TA004646-0293  (SNP7173) | add | 4A | 145.589 | -0.16 | -0.12 | 6.08 |
| RAC875_c67909_226  (SNP6451) | add | 4A | 151.228 | -0.17 | -0.12 | 6.11 |
| BS00059454_51  (SNP1327) | add | 4A | 151.675 | -0.17 | -0.12 | 6.16 |
| BobWhite_s66966_118  (SNP491) | add | 4A | 126.684 | 0.17 | -0.11 | 6.38 |
| Tdurum_contig93100_149  (SNP8084) | add | 4A | 136.506 | 0.17 | -0.11 | 6.52 |
| BS00091561_51  (SNP2089) | add | 4A | 136.506 | -0.17 | -0.11 | 6.61 |
| BobWhite_c8680_918  (SNP411) | add | 4A | 136.506 | -0.17 | -0.11 | 6.61 |
| Tdurum_contig7992_605  (SNP8013) | add | 4A | 126.684 | -0.17 | -0.11 | 6.64 |
| RAC875_rep_c104791_336  (SNP6611) | add | 4A | 144.773 | 0.17 | -0.12 | 6.67 |
| BobWhite_c14495_230  (SNP77) | add | 4A | 145.589 | -0.17 | -0.13 | 6.73 |
| wsnp_Ex_c4331_7808746  (SNP9082) | add | 4A | 126.684 | -0.17 | -0.12 | 6.96 |
| RFL_Contig4336_184  (SNP6925) | add | 4A | 105.209 | 0.16 | -0.14 | 7.01 |
| Kukri_c27648_350  (SNP4836) | add | 4A | 135.891 | 0.17 | -0.12 | 7.15 |
| BS00048067_51  (SNP1234) | add | 4A | 135.47 | 0.18 | -0.11 | 7.21 |
| IACX8322  (SNP4370) | add | 4A | 126.684 | -0.17 | -0.12 | 7.22 |
| BS00084703_51  (SNP2000) | add | 4A | 126.684 | 0.17 | -0.13 | 7.24 |
| wsnp_Ex_c33012_41567026  (SNP8988) | add | 4A | 161.192 | -0.16 | -0.14 | 7.26 |
| wsnp_CAP7_c254_138937  (SNP8477) | add | 4A | 137.211 | 0.18 | -0.12 | 7.44 |
| RAC875_c88582_131  (SNP6555) | add | 4A | 105.209 | 0.16 | -0.15 | 7.52 |
| Ex_c101546_376  (SNP2594) | add | 4A | 150.218 | 0.17 | -0.13 | 7.57 |
| RFL_Contig3841_1986  (SNP6906) | add | 4A | 137.308 | 0.18 | -0.12 | 7.62 |
| RFL_Contig3841_2595  (SNP6909) | add | 4A | 137.308 | 0.18 | -0.12 | 7.62 |
| wsnp_Ku_c20783_30448706  (SNP9772) | add | 4A | 137.697 | 0.18 | -0.12 | 7.64 |
| wsnp_Ex_c10955_17794520  (SNP8533) | add | 4A | 165.809 | 0.17 | -0.13 | 7.65 |
| RFL_Contig3841_2433  (SNP6908) | add | 4A | 138.998 | -0.18 | -0.13 | 7.96 |
| wsnp_Ku_c9746_16265584  (SNP9944) | add | 4A | 165.809 | -0.17 | -0.14 | 7.99 |
| RAC875_rep_c69632_65  (SNP6717) | add | 4A | 139.141 | 0.16 | -0.16 | 8 |
| wsnp_Ex_c8976_14964359  (SNP9354) | add | 4A | 165.809 | 0.17 | -0.15 | 8.01 |
| wsnp_Ex_rep_c67099_65575038  (SNP9480) | add | 4A | 165.809 | 0.18 | -0.13 | 8.01 |
| Excalibur_c33542_113  (SNP2996) | add | 4A | 139.141 | 0.16 | -0.16 | 8.18 |
| Tdurum_contig41127_265  (SNP7578) | add | 4A | 126.684 | 0.18 | -0.14 | 8.2 |
| RAC875_c95150_286  (SNP6577) | add | 4A | 113.554 | 0.18 | -0.14 | 8.29 |
| Tdurum_contig54776_1396  (SNP7828) | add | 4A | 165.809 | -0.17 | -0.15 | 8.41 |
| wsnp_Ex_c5072_9006966  (SNP9137) | add | 4A | 165.809 | 0.18 | -0.14 | 8.42 |
| BobWhite_c47168_289  (SNP332) | add | 4A | 149.752 | 0.18 | -0.15 | 8.45 |
| BobWhite_c25234_418  (SNP185) | add | 4A | 139.173 | 0.16 | -0.17 | 8.51 |
| wsnp_Ex_c2352_4405961  (SNP8857) | add | 4A | 113.392 | -0.17 | -0.17 | 8.56 |
| BobWhite_c25163_178  (SNP184) | add | 4A | 161.192 | 0.17 | -0.18 | 8.95 |
| BobWhite_c47168_598  (SNP333) | add/dom | 4A | 149.752 | -0.19 | -0.14 | 8.95 |
| Kukri_c48943_1149  (SNP5096) | add | 4A | 113.554 | -0.17 | -0.18 | 9.29 |
| Tdurum_contig46583_1275  (SNP7696) | add | 4A | 153.643 | -0.17 | -0.19 | 9.82 |
| BS00072157_51  (SNP1772) | add | 4A | 110.835 | 0.17 | -0.19 | 9.99 |
| Tdurum_contig46583_2203  (SNP7697) | add | 4A | 151.066 | -0.18 | -0.18 | 10.01 |
| RAC875_c4800_255  (SNP6269) | add | 4A | 153.009 | -0.17 | -0.2 | 10.03 |
| IACX5724  (SNP4300) | add | 4A | 153.009 | 0.17 | -0.2 | 10.15 |
| Excalibur_rep_c66939_849  (SNP3526) | add | 4A | 161.192 | -0.18 | -0.19 | 10.32 |
| GENE-0689_30  (SNP3641) | add | 4A | 136.506 | -0.16 | -0.21 | 10.34 |
| Excalibur_c46904_84  (SNP3127) | add | 4A | 153.009 | 0.17 | -0.2 | 10.35 |
| IAAV1383  (SNP3869) | add | 4A | 117.853 | 0.19 | -0.18 | 10.43 |
| BS00110021_51  (SNP2317) | add/dom | 4A | 139.173 | 0.17 | -0.21 | 10.44 |
| BS00039148_51  (SNP1122) | add | 4A | 113.392 | -0.17 | -0.21 | 10.66 |
| Tdurum_contig47476_528  (SNP7719) | add | 4A | 152.212 | -0.17 | -0.22 | 11 |
| Kukri_c20012_1425  (SNP4762) | add | 4A | 103.661 | -0.17 | -0.22 | 11.04 |
| Excalibur_rep_c112888_602  (SNP3514) | add/dom | 4A | 139.141 | -0.16 | -0.24 | 11.22 |
| Kukri_rep_c85536_598  (SNP5519) | add | 4A | 161.691 | 0.17 | -0.23 | 11.27 |
| Tdurum_contig47476_495  (SNP7718) | add | 4A | 152.31 | 0.17 | -0.22 | 11.35 |
| BobWhite_c11327_185  (SNP27) | add/dom | 4A | 137.697 | 0.17 | -0.23 | 11.83 |
| BobWhite_c20306_88  (SNP144) | add/dom | 4A | 139.173 | -0.17 | -0.23 | 11.87 |
| Tdurum_contig45738_670  (SNP7687) | add/dom | 4A | 138.998 | 0.16 | -0.25 | 12 |
| RFL_Contig2531_1872  (SNP6846) | add/dom | 4A | 149.752 | 0.17 | -0.25 | 12.31 |
| tplb0057f21_914  (SNP8254) | add | 7D | 7.732 | 0.17 | -0.21 | 10.3 |
| BS00099983_51  (SNP2209) | add | Unmapped | NA | 0.17 | -0.11 | 6.76 |
| CAP7_c254_486  (SNP2444) | add | Unmapped | NA | 0.17 | -0.11 | 7.11 |
| Jagger_rep_c10298_150  (SNP4431) | add/dom | Unmapped | NA | -0.17 | -0.22 | 11.08 |

**Suppl. Table 4:** Effects of detected significant markers on stripe rust resistance, their chromosomal (Chrom.) position (Pos.), the additive (Add.) and dominance (Dom.) effects, and the adjusted (Adj.) R².

| **Marker (SNP)** | **Type** | **Chrom.** | **Pos. (cM)** | **Add. effect** | **Dom. effect** | **Adj. R^2^ (%)** |
| --- | --- | --- | --- | --- | --- | --- |
| RAC875_rep_c107961_348  (SNP6642) | dom | 2A | 96.188 | 0.51 | -0.12 | 3.34 |
| CAP12_c259_307  (SNP2402) | add | 2A | 21.441 | -0.21 | -0.16 | 4.79 |
| IACX11417  (SNP4235) | add/dom | 2A | 21.441 | 0.22 | -0.14 | 4.97 |
| wsnp_RFL_Contig1951_1127302  (SNP10123) | add/dom | 2A | 21.902 | -0.22 | -0.14 | 5.14 |
| RAC875_c829_1143  (SNP6526) | add/dom | 2A | 15.441 | 0.23 | -0.14 | 5.58 |
| BS00076693_51  (SNP1862) | add | 2A | 29.121 | 0.36 | -0.21 | 5.74 |
| RAC875_c26214_505  (SNP6000) | add/dom | 2A | 21.521 | 0.24 | -0.15 | 5.74 |
| RAC875_c47161_100  (SNP6262) | add | 2A | 21.829 | 0.24 | -0.14 | 6.17 |
| BS00022532_51  (SNP816) | add/dom | 2A | 15.441 | 0.25 | -0.14 | 6.45 |
| tplb0041b11_529  (SNP8217) | add/dom | 2A | 15.441 | 0.25 | -0.18 | 6.91 |
| D_contig01272_220  (SNP2512) | add/dom | 2A | 22.407 | -0.26 | -0.16 | 6.98 |
| Tdurum_contig11802_864  (SNP7369) | add/dom | 2A | 15.441 | 0.27 | -0.17 | 7.63 |
| Kukri_c23195_266  (SNP4786) | add/dom | 2A | 15.441 | 0.28 | -0.17 | 7.86 |
| Kukri_c31776_1621  (SNP4907) | add/dom | 2A | 15.441 | 0.27 | -0.16 | 7.87 |
| Tdurum_contig29983_490  (SNP7535) | add/dom | 2A | 14.444 | 0.28 | -0.17 | 7.98 |
| Excalibur_c18324_390  (SNP2798) | add/dom | 2A | 15.441 | 0.28 | -0.16 | 8.15 |
| BobWhite_c12426_84  (SNP49) | add/dom | 2A | 15.441 | 0.28 | -0.16 | 8.15 |
| Excalibur_c25599_358  (SNP2903) | add/dom | 2A | 15.441 | 0.28 | -0.17 | 8.21 |
| Kukri_c12648_434  (SNP4669) | add/dom | 2A | 15.441 | -0.29 | -0.16 | 8.26 |
| Tdurum_contig63196_123  (SNP7943) | add/dom | 2A | 15.441 | 0.28 | -0.14 | 8.32 |
| IACX6178  (SNP4341) | add/dom | 2A | 21.441 | 0.28 | -0.15 | 8.51 |
| wsnp_Ku_c23598_33524490  (SNP9787) | dom | 2A | 7.065 | -0.4 | -0.17 | 9.25 |
| BobWhite_c13373_250  (SNP59) | dom | 2A | 7.41 | -0.42 | -0.14 | 10.78 |
| wsnp_Ex_c2772_5130007  (SNP8923) | add/dom | 2A | 2.886 | 0.36 | -0.24 | 10.93 |
| BS00068050_51  (SNP1675) | dom | 2A | 22.272 | 0.48 | -0.23 | 11.01 |
| Excalibur_c21663_145  (SNP2847) | add/dom | 2A | 15.441 | 0.43 | -0.24 | 11.77 |
| RFL_Contig174_406  (SNP6824) | add | 2A | 3.612 | 0.44 | -0.24 | 12.03 |
| wsnp_Ex_c19516_28481857  (SNP8770) | add/dom | 2A | 3.612 | -0.46 | -0.25 | 12.07 |
| TA002254-0660  (SNP7071) | add/dom | 2A | 15.441 | -0.41 | -0.23 | 15.83 |
| BobWhite_c16735_131  (SNP100) | dom | 2B | 90.065 | -0.3 | -0.13 | 5.16 |
| IAAV1743  (SNP3890) | dom | 2B | 90.084 | 0.31 | -0.13 | 5.54 |
| Jagger_c7212_85  (SNP4423) | dom | 2B | 90.084 | 0.31 | -0.13 | 5.54 |
| Ra_c6266_140  (SNP5652) | dom | 2B | 90.065 | 0.31 | -0.13 | 5.54 |
| wsnp_JD_c9251_10121369  (SNP9665) | dom | 2B | 90.065 | -0.31 | -0.13 | 5.63 |
| BobWhite_c10215_242  (SNP2) | dom | 2B | 90.117 | 0.31 | -0.13 | 5.68 |
| RAC875_c1226_652  (SNP5741) | dom | 2B | 78.215 | -0.28 | -0.19 | 8.01 |
| BobWhite_c17047_268  (SNP109) | add | 2B | 13.177 | -0.44 | -0.26 | 8.69 |
| RAC875_c5577_1682  (SNP6348) | add/dom | 2B | 15.12 | 0.4 | -0.23 | 10.92 |
| BS00093111_51  (SNP2113) | add/dom | 2D | 11.927 | 0.27 | -0.16 | 7.53 |
| BS00093990_51  (SNP2132) | add/dom | 2D | 23.98 | -0.29 | -0.17 | 8.56 |
| IAAV5607  (SNP4060) | dom | 4D | 101.799 | -0.38 | -0.07 | 1.55 |
| BS00040814_51  (SNP1156) | dom | 6A | 133.294 | -0.08 | -0.12 | 1.37 |
| Tdurum_contig29607_413  (SNP7531) | dom | 6A | 122.844 | -0.19 | -0.04 | 2.31 |
| RAC875_c8721_212  (SNP6544) | dom | 6A | 133.294 | -0.17 | -0.09 | 2.68 |
| wsnp_RFL_Contig4456_5258284  (SNP10160) | dom | 6A | 129.726 | 0.18 | -0.08 | 2.72 |
| Jagger_c5046_63  (SNP4409) | dom | 6A | 133.294 | -0.17 | -0.08 | 2.74 |
| GENE-4021_496  (SNP3791) | dom | Unmapped | NA | -0.17 | -0.08 | 2.56 |
| RAC875_c829_1215  (SNP6527) | add/dom | Unmapped | NA | 0.22 | -0.14 | 4.99 |
| Ra_c6266_136  (SNP5651) | dom | Unmapped | NA | 0.31 | -0.13 | 5.54 |
| Excalibur_c62893_629  (SNP3256) | add/dom | Unmapped | NA | 0.24 | -0.14 | 6.08 |
| BS00067382_51  (SNP1627) | add/dom | Unmapped | NA | 0.27 | -0.18 | 7.03 |
| TA003766-0683  (SNP7139) | add/dom | Unmapped | NA | -0.27 | -0.15 | 7.04 |
| Kukri_c22599_114  (SNP4782) | add/dom | Unmapped | NA | -0.26 | -0.17 | 7.28 |
| RAC875_rep_c113106_93  (SNP6677) | add/dom | Unmapped | NA | 0.27 | -0.17 | 7.49 |
| IAAV8501  (SNP4180) | add/dom | Unmapped | NA | -0.27 | -0.16 | 7.75 |
| Ra_c26532_894  (SNP5607) | add/dom | Unmapped | NA | 0.27 | -0.16 | 7.92 |


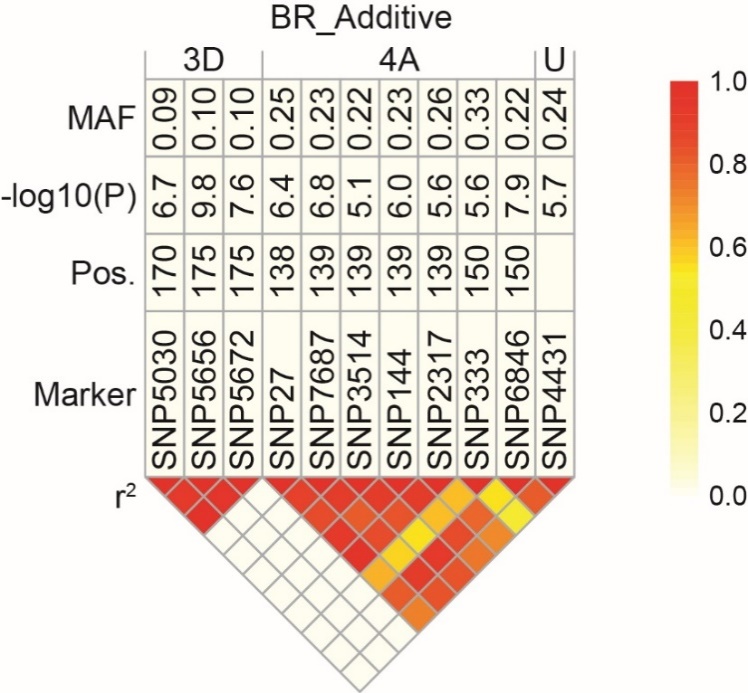


**Suppl. Figure 4:** SNPs with additive effects for leaf rust resistance. Table including minor allele frequency (MAF), significance value (-log 10(P)), and genetic map position of respective SNP markers that contribute significantly to the additive genetic variation of leaf rust resistance. The heat plot presents the linkage disequilibrium (LD) measured as squared Pearson’s correlation coefficients (r²) among SNP markers.

**Suppl. Figure 5:** SNPs with additive effects for stripe rust resistance. Table including minor allele frequency (MAF), significance value (-log 10(P)), and genetic map position of respective SNP markers that contribute significantly to the additive genetic variation of stripe rust resistance. The heat plot presents the linkage disequilibrium (LD) measured as squared Pearson’s correlation coefficients (r²) among SNP markers.


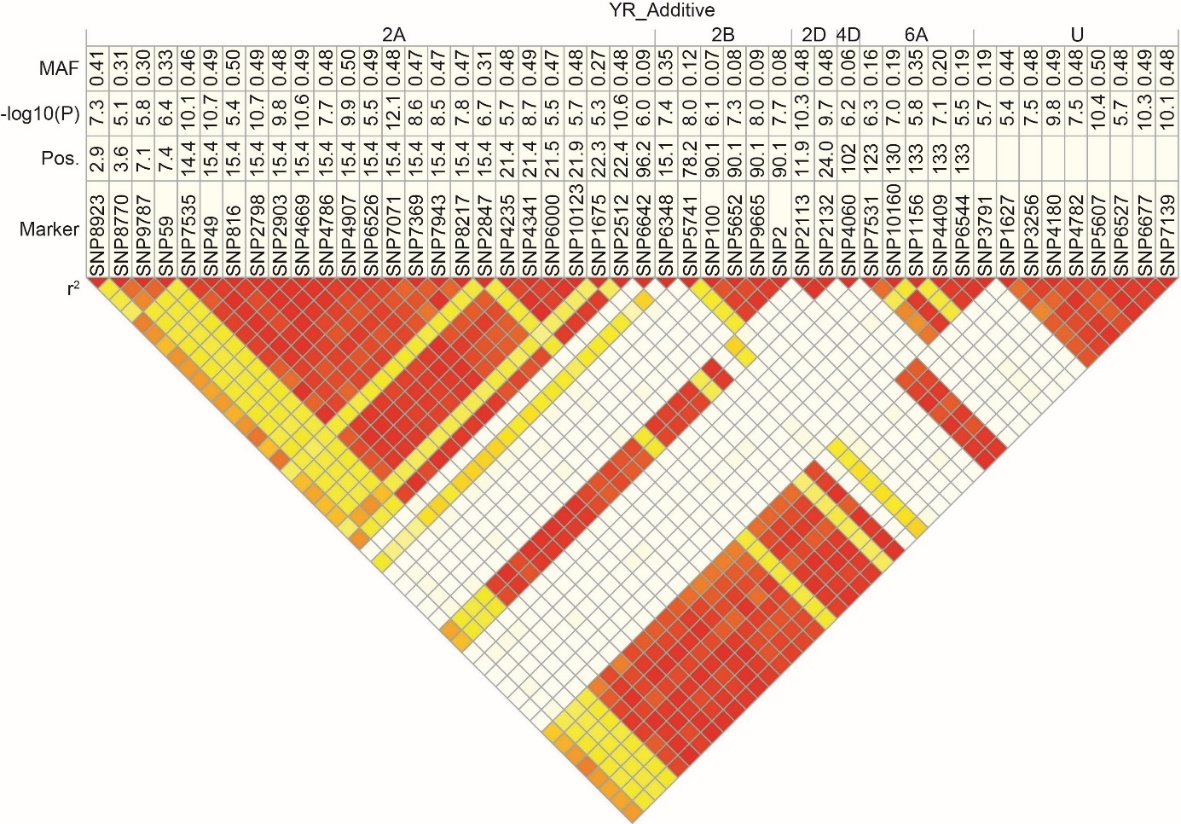

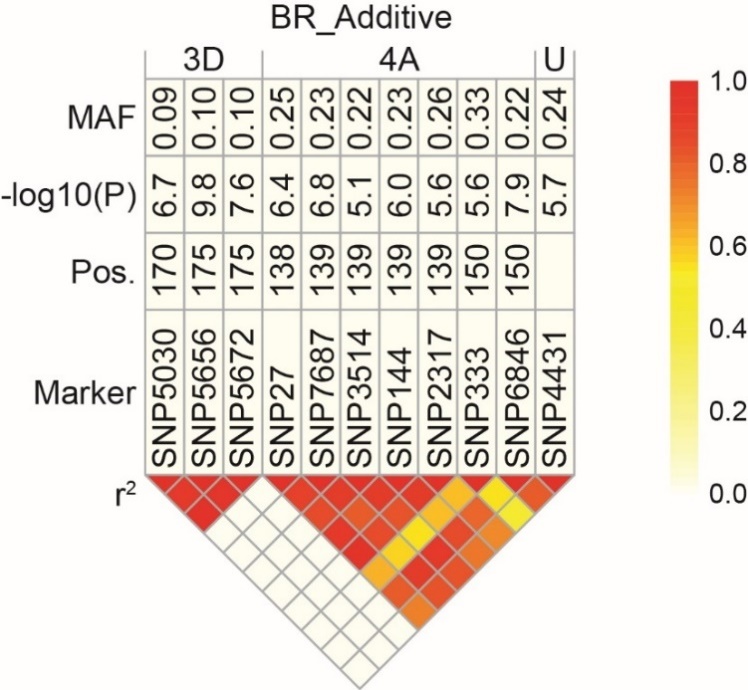

Supplement: Supplementary file 1 — Supplementary material 1 (DOCX 4505 kb) [file 122_2020_3588_MOESM1_ESM.docx]
